# Psychometric properties of the Dating Violence Questionnaire for Victimization and Perpetration (DVQ-VP) in Ecuadorian population

**DOI:** 10.1186/s41155-025-00359-w

**Published:** 2025-08-21

**Authors:** Andrés Ramírez, Luis Burgos-Benavides, Javier Herrero Díez, Hugo Sinchi-Sinchi, Alhena L. Alfaro-Urquiola, Venus Medina-Maldonado, Francisco Javier Rodríguez-Díaz

**Affiliations:** 1 Department of Clinical Psychology. Grupo de Investigación en Neurociencia Clínica Aplicada (GINCA), Universidad Politécnica Salesiana, Cuenca, Ecuador; 2 Department of Psychology, University of Oviedo, Oviedo, Spain; 3 Department of Psychology, Pontificia Universidad Católica del Ecuador, Esmeraldas, Ecuador; 4 Universidad Católica Boliviana “San Pablo”, La Paz, Bolivia; 5 Department of Nursing, Gender-Based Violence Prevention Research Group (E-previo), Faculty of Health and Wellbeing, Centro de Investigación para la Salud de América Latina (CISeAL), Pontifical Catholic University of Ecuador, Quito, Ecuador

**Keywords:** Adaptation, Psychometrics, Validation, Dating violence

## Abstract

**Objective:**

The aim of this study was to evaluate the psychometric properties of the Dating Violence Questionnaire for Victimization and Perpetration (DVQ-VP) in a sample of the Ecuadorian population.

**Methods:**

The study included 819 participants (47% men and 53% women), consisting of Ecuadorian adolescents and university students. An instrumental design was employed for the linguistic adaptation, reliability, and convergent (AVE), discriminant (HTMT), and structural (CFI, TLI, and RMSEA) validation of the DVQ-VP. The construct validity and internal consistency of the instrument were assessed. Construct validity was analyzed using confirmatory factor analysis (CFA), while internal consistency was evaluated using ordinal Cronbach’s alpha and McDonald’s omega coefficients. Additionally, a network analysis was conducted with the DVQ-VP items.

**Results:**

The CFA results indicated that the DVQ-VP has a factorial structure consistent with the original theoretical model, with adequate fit indices (CFI > 990, TLI > 990, and RMSEA < 0.08 in both models of the DVQ-VP). The Cronbach’s alpha and McDonald’s omega values for the victimization and perpetration subscales were above .70, indicating good internal consistency. Additionally, the instrument proved to be sensitive in identifying different forms of dating violence.

**Conclusion:**

The findings support that the DVQ-VP demonstrated adequate levels of validity and reliability for assessing dating violence in a sample of Ecuadorian adolescents and university students. These results suggest that, within this specific context and population, the instrument can be a useful tool for identifying patterns of victimization and perpetration in dating relationships. Its application may contribute to early detection and the development of targeted interventions to reduce intimate partner violence among young people.

## Introduction

Intimate partner violence (IPV) is a widespread social and health problem affecting millions of people worldwide (Ince-Yenilmez 2022; Sardinha et al., 2022). It encompasses physical, emotional, and sexual abuse, and its impact extends to both immediate and long-term health and social consequences (Cotter, 2021). To address IPV effectively, it is crucial to use comprehensive resources and tools, starting from precise measurement to primary prevention strategies (Davis, and Padilla-Medina, 2021). This enables a better understanding and more effective intervention (Campbell et al., 2024).

Psychometric tests, alongside interviews, are fundamental instruments used by psychologists to measure, analyze, and understand human behavior (Lee & Wong, 2020). These tests are extensively used in applied research to quantify IPV accurately (Gómez-Fernández et al., 2019). Over the years, various measurement instruments have been developed and validated in scientific literature, each tailored to different aspects and populations affected by Intimate partner violence (Sereno et al., 2024). Specifically, some instruments focus on young populations, reflecting the unique dynamics of intimate relationships in this age group (Jennings et al., 2017).

Intimate partner violence in these populations is often referred to as dating violence (DV, Evans et al., 2022). Dating violence generally includes any form of abuse within a romantic relationship among adolescents and young adults who are not cohabiting or married. However, the definition of dating violence can vary considerably (Arrojo et al., 2024; Duval et al., 2018; Marcos et al., 2023). For the purposes of this study, DV is defined following the approach of Rodríguez Díaz et al. (2017), Rodríguez Franco et al. (2017), Knopp et al. (2020), and Aguilera Jiménez et al. (2021). This definition excludes relationships involving married or cohabiting partners, as well as those with legal formalities. The presence of children in such relationships is also considered a factor in exclusion, as these dynamics may align more closely with Intimate partner violence in adult populations rather than dating violence among adolescents or young adults (Lee & Wong, 2020). By clarifying these parameters, this study ensures a more precise understanding of the types of relationships and the scope of dating violence being investigated (Riesgo-González, et al., 2019).

This leads Escoto et al. (2007) to consider that adolescents create great expectations of love, care, support, and understanding in courting, which prevents them from noticing when they become involved in dating violence. This idealization can obscure their perception of abusive behaviors, making it difficult for them to recognize the signs of dating violence (Kennedy et al., 2021). Consequently, this leads to a great diversity of definitions, ideologies, and methodological positions in the different groups and contexts in which dating violence has been investigated (Padilla-Medina et al., 2022).

The importance of studying this phenomenon lies in its predictive nature of future aggression, something that will become increasingly serious with time and coexistence (Martínez-Gómez et al., 2021). Understanding dating violence in adolescence is crucial for developing effective preventive measures and interventions, as early experiences of violence can set a precedent for future abusive relationships (Espelage et al., 2020). The complexity and variability in how dating violence is defined and studied underscore the need for a comprehensive approach to addressing this issue, considering the unique dynamics of young relationships and the long-term implications for individuals involved (Campo-Tena et al., 2024).

It is understood that dating violence encompasses any act, omission, attitude, or expression that generates, or has the potential to generate, emotional, physical, or sexual harm to the affective partner with whom an intimate relationship is maintained in the absence of cohabitation, economic bond, legal, or marital status (Toplu-Demirtaş et al., 2022). This form of violence has specific characteristics not shared with the violence experienced in other stages of a couple’s life (Rubio-Garay et al., 2019).

As a social problem, dating violence, from a legal perspective and linked to the intervention of power, must imply intentionality, not be desired, not be essential, and be harmful (Hamby, 2017). This necessitates discriminating the perception of the behavior to determine whether it is an isolated act or part of a process (García-Díaz et al., 2020; López-Cepero et al., 2015).

A notable characteristic of this type of violence is its bidirectional nature, meaning it can be perpetrated by either partner, regardless of their sex, especially in cases of psychological and physical violence (Capinha et al., 2022). Research indicates that as the level of aggressiveness increases, this bidirectional characteristic diminishes, with more women being affected as the relationship becomes more stable (Herrero Olaizola, et al., 2020; Rey-Anacona et al., 2021; Riesgo-Gonzalez et al., 2019).

Furthermore, adolescents often create great expectations of love, care, support, and understanding in courting, which prevents them from noticing when they become involved in dating violence (Lilly et al., 2023). This idealization can obscure their perception of abusive behaviors, making it difficult for them to recognize the signs of dating violence. Consequently, this leads to a great diversity of definitions, ideologies, and methodological positions in the different groups and contexts in which dating violence has been investigated.

The importance of studying this phenomenon lies in its predictive nature of future aggression, something that will become increasingly serious with time and coexistence (Rey-Anacona et al., 2021). Understanding dating violence in adolescence is crucial for developing effective preventive measures and interventions, as early experiences of violence can set a precedent for future abusive relationships. The complexity and variability in how dating violence is defined and studied underscore the need for a comprehensive approach to addressing this issue, considering the unique dynamics of young relationships and the long-term implications for individuals involved. This approach, in addition to making its identification more precise and improving its surveillance, will promote a better evaluation and may offer a better guide to its treatment (Hamby, 2017). This implies the need for effective instruments aimed at evaluating the presence of violence in couple relationships, either those aimed at diagnosing abuse within the couple/dating violence (long instruments) or those that seek to make a detection orientation or screen (Pineda et al., 2023).

However, many of these instruments lack methodological rigor in their validation process. Among those used with Spanish-speaking samples, three stand out: the Conflict Tactics Scale 2 (CTS2), the Conflict in Adolescent Dating Relationships Inventory (CADRI), and the Dating Violence Questionnaire (DVQ) (López-Cepero et al., 2015). The CTS2 is noted for not being adequately prepared for working with young people and adolescents, and the CADRI is criticized for not delving deeply into the study of victimization, in addition to retaining Anglo-Saxon elements that may not adapt well to other cultural contexts (Ramírez et al., 2025a).

The Dating Violence Questionnaire is proposed as the most appropriate tool to study this topic, especially with its revised and reduced version, the Dating Violence Questionnaire for Victimization and Perpetration (DVQ-VP) (Rodríguez-Díaz, et al., 2017; Rodríguez-Franco, et al., 2022). The DVQ-VP offers a culturally adapted, methodologically rigorous instrument that addresses the specific needs of evaluating dating violence among Spanish-speaking populations. Its development involved extensive validation processes to ensure reliability and accuracy, making it a valuable tool for both research and clinical applications.

In this study, the DVQ-VP was used as the primary instrument to assess dating violence, given its utility and structure in addressing the various dimensions of this phenomenon (Rodríguez-Franco, et al., 2022). However, it is recommended that future research consider using other instruments, such as the Conflict in Adolescent Dating Relationship Inventory (CADRI) or the Multidimensional Scale Dating Violence (MSDV). Employing different tools could provide complementary perspectives, enhance the comparability of results, and evaluate cultural sensitivity, validity, and reliability across diverse populations. This would contribute to a more comprehensive and robust understanding of dating violence.

It is important to note that most psychometrically supported tests have been developed and validated with samples from the USA or Europe. This can lead to overlooking the cultural component and sociodemographic context as crucial factors in the phenomenon being studied (Fernández et al., 2010). This gap underscores the necessity for culturally adapted instruments to ensure precise and relevant assessments across various populations. In this context, the original version of the DVQ-VP was specifically developed for the Latin American population, making it an essential tool for this demographic. The DVQ-VP now has a version that examines the phenomenon from both the victim’s and the aggressor’s perspectives (Alfaro-Urquiola et al., 2024; Rodríguez-Franco et al., 2022). This dual approach is vital for a thorough understanding of the dynamics of dating violence.

The main objective of this study is to evaluate the psychometric properties of the DVQ-VP in a sample of the Ecuadorian population. This purpose involves validating the instrument to ensure it accurately reflects the characteristics of dating violence as experienced by adolescents and young adults in Ecuador.

The first specific objective is to determine the reliability of the DVQ-VP by evaluating the internal consistency and temporal stability of the victimization (DVQ-RV) and perpetration (DVQ-RP) subscales, specifically adapted to the Ecuadorian context. This will allow for an assessment of the instrument’s reliability in identifying dating violence in this population. Furthermore, the study seeks to validate the five-dimensional model of the DVQ-VP (physical violence, psychological violence, sexual violence, controlling behaviors, and relational aggression) within the Ecuadorian context through factor analysis to confirm that the identified dimensions remain consistent within the local population. A third objective is to analyze and estimate the psychometric parameters of each factor of the DVQ-VP, such as factor loadings and explained variance, to confirm that the five-dimensional model is suitable and robust for the Ecuadorian population.

Additionally, the study aims to evaluate the convergent and discriminant validity of the DVQ-VP, meaning that the subscales of the instrument should positively correlate with other measures related to dating violence and clearly distinguish from instruments that do not address this issue, confirming its specificity and usefulness within the Ecuadorian population. A network analysis of the DVQ-VP items will also be conducted to identify significant relationships between the victimization and perpetration dimensions, providing a deeper understanding of how different forms of dating violence interact within the Ecuadorian population.

Finally, the correlations between the victimization and perpetration subscales of the DVQ-VP will be examined, with the expectation that these subscales will be moderately correlated, indicating that both dimensions are interrelated but maintain their distinction, allowing for a precise assessment of the experience and perpetration of dating violence.

It is hypothesized that the DVQ-VP will demonstrate high reliability in both subscales (victimization and perpetration) in the Ecuadorian population, indicating that the instrument is consistent and reliable for measuring dating violence. Additionally, it is expected that the DVQ-VP will adequately fit a five-dimensional model (physical violence, psychological violence, sexual violence, controlling behaviors, and relational aggression), validating the theoretical structure of the instrument in the Ecuadorian context.

It is hypothesized that the estimated parameters for the factors of the DVQ-VP, such as factor loadings and explained variance, will be statistically significant, indicating that the proposed factor model is appropriate and robust for the Ecuadorian population. Furthermore, it is expected that the DVQ-VP will demonstrate convergent validity by positively correlating with other measures related to dating violence and discriminant validity by not correlating excessively with measures that do not address this issue, confirming its specificity and usefulness for the Ecuadorian population.

It is hypothesized that the network analysis will reveal significant relationships between the dimensions of victimization and perpetration, contributing to a more detailed understanding of the interactions between the different forms of dating violence in Ecuador. Finally, it is hypothesized that the subscales of victimization and perpetration in the DVQ-VP will be moderately correlated, indicating that both dimensions are interrelated but maintain their distinction, allowing for an accurate assessment of dating violence in the Ecuadorian population.

## Methods

### Design

An instrumental study was conducted in two phases to evaluate the psychometric properties of the Dating Violence Questionnaire for Victimization and Perpetration (DVQ-VP) in the Ecuadorian population. In the first phase, a linguistic adaptation of the original questionnaire was performed through an iterative translation process by experts (Arias & Sireci, 2021). In the second phase, evidence of reliability and validity for the adapted version of the DVQ-VP in Ecuador was examined, following current standards for the validation of psychological tests, as well as guidelines for the adaptation and translation of existing tests (Muñiz et al., 2013).

### Participants

The study successfully included a total of 819 participants, representing a considerable sample size that allows for exploring trends and characteristics of the phenomenon under investigation. The equitable gender distribution, with 47% men and 53% women, ensures balanced representation, facilitating gender-differentiated analyses and enriching the interpretation of the results. Although a non-probabilistic convenience sampling method was used, this approach is useful in exploratory studies (Etikan & Bala, 2017). Furthermore, the sample size is adequate to ensure more than 10 subjects per item of the instrument used, which reinforces the statistical stability of the analyses and enhances the internal reliability of the findings.

In terms of ethnicity, the vast majority of participants identified as mestizos, with 741 individuals constituting 90% of the sample. Other ethnic groups were represented in smaller proportions: there were 10 montubios (1.2*%*), 11 white individuals (1.3*%*), 5 Ecuadorian indigenous people (0.6%), and 52 Afro-Ecuadorians (6.3%).

The descriptive analysis of the sample’s age by sex showed that the mean age for men was 20.7 years and for women was 20.5 years, with an overall mean of 20.6 years. The median age was 21 years for men and 20 years for women, with a total median of 21 years. The standard deviation (SD) was 2.64 for men and 2.38 for women, with an overall standard deviation of 2.51. The minimum age in the sample was 15 years for both sexes, and the maximum age was 25 years for both sexes as well. The first quartile (*Q*_1_) for both sexes was 19 years. The second quartile (*Q*_2_) or median was 21 years for men and 20 years for women. The third quartile (*Q*_3_) was 23 years for men and 22 years for women, with an overall *Q*_3_ of 23 years (Table 1).

**Table 1:** Descriptive analysis of the age of the sample according to sex

	**Man** (*n* = 388)	**Woman** (*n* = 431)	**Total** (*n* = 819)
*Media*	20.7	20.5	20.6
*Median*	21	20	21
*Standard Deviation*	2.64	2.38	2.51
Minimum	15	15	15
Minimum	25	25	25
*Q*_1_ (*25th*)	19	19	19
*Q*_2_ (*50th*)	21	20	21
*Q*_3_ (*75th*)	23	22	23

### Instruments

Ad Hoc Sociodemographic Data Survey: A custom form was administered to collect sociodemographic data such as age, sex, and ethnicity.

Dating Violence Questionnaire for Victimization and Perpetration (DVQ-VP): Developed by Rodríguez Franco et al. (2022), is a comprehensive instrument designed to assess dating violence from a bidirectional perspective. This single questionnaire consists of two complementary dimensions which, rather than being separate tools, are integrated components of the same measure: the victimization dimension (DVQ-RV) and the perpetration dimension (DVQ-RP). Both dimensions share an identical five-factor correlated structure, allowing for the examination of different forms of dating violence: physical, sexual, humiliation, detachment, and coercion. The victimization dimension evaluates experiences of violence received from the current partner through 20 items, such as “Has your partner hit you?” or “Does your partner insist on touching you in ways or places you don’t like or want?”, using a five-point Likert scale ranging from 0 (never) to 4 (all the time). The measurement model for the DVQ-RV was calculated based on this correlated factor structure, with all items significantly loading onto their respective factors and demonstrating good model fit: χ^2^(160) = 345.24, *p* ≤ 0.001, CFI = 0.973, RMSEA = 0.031, 90% *CI* [0.026, 0.035]. Likewise, the perpetration dimension adapts the same items to assess aggressive behaviors directed at the partner, with questions such as: “Have you hit your partner?” or “Do you insist on touching your partner in ways or places they don’t like or want?”. The measurement model for the DVQ-RP was based on the same five-factor correlated structure as the victimization model, with all items significantly loading onto their respective factors and a good model fit observed: χ^2^(160) = 312.38, *p* ≤ 0.001, CFI = 0.967, RMSEA = 0.028, 90% *CI* [0.023, 0.032].

### Linguistic equivalence of the DVQ-VP

Although the original Dating Violence Questionnaire (DVQ) was developed in Spain (Rodríguez-Franco et al., 2022), an English version was later generated to support its use in international research contexts. In this study, the English version was used as the reference point to ensure semantic and conceptual alignment with other cross-cultural validations. Instead of directly adapting the European Spanish version, the team followed a triangulated process that included both the English version and the original Spanish source, with the goal of achieving a version that was culturally and linguistically appropriate for the Ecuadorian population.

The adaptation process followed an iterative methodology led by experts. Two independent translations from English to Spanish were produced by bilingual professionals, and a third version was derived directly from the Spanish (Spain) version of the DVQ-VP. These three versions were compiled into a review booklet, which was evaluated by a panel of three bilingual experts one linguist and two psychologists specialized in dating violence. Items were assessed for semantic equivalence, clarity, and linguistic familiarity specific to Ecuadorian Spanish. The item order was counterbalanced to avoid bias, and experts were invited to propose additional suggestions. This approach ensured both conceptual integrity and contextual relevance for the Ecuadorian setting.

In the third stage, the experts’ responses were independently compared to identify discrepancies. In the fourth stage, the authors reached a consensus on the final version of each item. Subsequently, this consensual version was reviewed by a group of university students, who provided feedback that allowed for minor adjustments in grammar and meaning. It is important to note that none of the participants involved in this preliminary process participated in subsequent studies on the instrument’s reliability and validity.

### Procedure

First, the back-translation method was employed to ensure the semantic and conceptual equivalence of the instruments used. Although the original Dating Violence Questionnaire (DVQ) was developed in Spanish (Rodríguez-Franco et al., 2022), an intermediate English version was used as the reference for translation into Ecuadorian Spanish, aiming to maintain consistency with international research applications. This process included a thorough cultural adaptation to ensure the instrument’s relevance and comprehensibility within the Ecuadorian context. Bilingual translators and subject matter experts in dating violence evaluated the items for fidelity to the original construct, clarity, and appropriateness of everyday language, ensuring the instrument’s validity for use in this specific population.

After informing the participants about the study’s objectives, signed informed consent forms were collected from participants aged 15 and older, as well as from the parents or legal guardians of minors. The instruments were administered collectively (in person) and anonymously in a single session held at the participants’ respective educational institutions. Minor students without parental consent were relocated to another classroom to engage in school-determined activities.

To standardize the administration of the instruments, the responsible psychologists used a written protocol outlining the instructions to be followed and the administration conditions, tailored to different age groups. The order of the questionnaires was as follows: first, the sociodemographic questionnaire, followed by the Spanish version of the Dating Violence Questionnaire for Victims and Perpetrators (DVQ-VP). The study was approved (code 046-UIO-2022) by the Human Research Ethics Committee of the Pontifical Catholic University of Ecuador.

### Statistical analysis

The analyses were performed using R software version 4.1 (R Core Team., 2022) and Jamovi version 2.6.26. Prior to conducting factor analysis, we first carried out a data screening to evaluate data distribution and verify the assumptions necessary for factor analysis. We assessed multivariate normality using Mardia’s test and checked these assumptions by examining the residuals from a linear regression applied to a set of random numbers. Any anomalies in the distribution of residuals from a spurious regression were attributed to the data itself.

To evaluate the structural validity of the scales, we conducted a confirmatory factor analysis (CFA) with the R package *lavaan* (Rosseel, 2012). Given the lack of multivariate normality in the data, we employed a diagonally weighted least squares estimator. Data reliability was assessed using Cronbach’s (α) and McDonald’s (ω) (Revelle, 2017).

For evaluating convergent and discriminant validity, we used the *SemTools package* (Jorgensen et al., 2022). Convergent validity was determined through the average variance extracted (AVE), which needed to be at least 0.37 (Moral de la Rubia., 2019; Ramírez et al., 2025a, 2025b). Discriminant validity was assessed by calculating the heterotrait-monotrait ratio (HTMT), with a threshold below 0.85 indicating acceptable discriminant validity (Henseler et al., 2015). Additionally, we conducted a network analysis of the victimization and perpetration items to assess external validity (Golino & Christensen, 2021).

The statistical analysis also involved evaluating the fit of two models Victimization and Perpetration for the Ecuadorian version. We used various fit indices, including the Comparative Fit Index (CFI), Tucker-Lewis Index (TLI), Bentler-Bonett Non-normed Fit Index (NNFI), Relative Noncentrality Index (RNI), Bentler-Bonett Normed Fit Index (NFI), Bollen’s Relative Fit Index (RFI), and Bollen’s Incremental Fit Index (IFI). Additionally, we calculated the root mean square error of approximation (RMSEA) and its 95% confidence intervals to further assess the models’ fit (Lubbe, 2023; Ramírez et al., 2025b).

## Results

The results of the descriptive analysis and reliability of the DVQ-VP questionnaire items, which measure victimization and perpetration in the context of dating violence, provide a detailed view of the response distribution and the internal consistency of the scale (Table 2).

**Table 2:** Reliability of the Victimization (DVQ-RV) and Perpetration (DVQ-RP) models Ecuadorian version

	**M**	**SD**	**α**	**ω**
Victimization	0.243	0.273	.827	.867
Perpetration	0.252	0.307	.847	.874

*M *Media, *SD *Standard Deviation, *α *Alpha Ordinal, *ω *McDonald’s

In the victimization subscale, participants reported on average, a low frequency of victimization experiences, with a mean of 0.243 and a standard deviation of 0.273. The response distribution shows high skewness and kurtosis in several items, indicating that most participants reported few or no victimization experiences, with very few reporting more extreme incidents. The item correlations with the rest of the scale vary, suggesting a moderate relationship among the items contributing to the victimization construct. The subscale’s reliability, measured through the ordinal Cronbach’s alpha and McDonald’s omega coefficient, is high, indicating that the items reliably measure the concept of victimization.

Similarly, the perpetration subscale also reflects a low frequency of perpetration behaviors among participants, with a mean of 0.252 (*SD* = 0.307). As with victimization, the items show a distribution skewed toward low values, with few extreme cases. The item correlations in this subscale are similar to those in the victimization subscale, indicating moderate relationships among the items. The internal consistency of the subscale is high, as reflected by both the ordinal alpha and the omega coefficient, suggesting that this subscale is also a reliable tool for measuring perpetration behaviors in dating violence.

Overall, these results show that while the experiences of victimization and perpetration are generally low, the scales used to measure these constructs exhibit high reliability, making them suitable instruments for assessing these dimensions in the studied context.

In the Ecuadorian version of the Victimization (DVQ-RV) and Perpetration (DVQ-RP) models, the fit indices show very favorable results. Model 1, corresponding to Victimization (DVQ-RV), presents a Comparative Fit Index (CFI) of 0.995, a Tucker-Lewis Index (TLI) of 0.993, a Bentler-Bonett Non-normed Fit Index (NNFI) of 0.993, and a Relative Noncentrality Index (RNI) of 0.995. Additionally, the Bentler-Bonett Normed Fit Index (NFI) is 0.982, Bollen’s Relative Fit Index (RFI) is 0.976, and Bollen’s Incremental Fit Index (IFI) is 0.995. The root mean square error of approximation (RMSEA) for this model is 0.02, with a 95*%* confidence interval ranging from 0.012 to 0.031 (Table s3).

In contrast, Model 2, corresponding to Perpetration (DVQ-RP), also shows good fit indices, though slightly lower compared to Model 1. The CFI is 0.992, TLI is 0.991, NNFI is 0.991, and RNI is 0.992. The NFI for this model is 0.976, RFI is 0.971, and IFI is 0.992. The RMSEA for Model 2 is 0.023, with a 95*%* confidence interval ranging from 0.016 to 0.030 (Table 3). These indices indicate that both models have an excellent fit to the data, suggesting that the Ecuadorian versions of the DVQ-RV and DVQ-RP instruments are suitable for measuring victimization and perpetration of violence in the Ecuadorian context.

**Table 3:** Victimization (DVQ-RV) and Perpetration (DVQ-RP) models Ecuadorian version

	**Model 1**	**Model 2**
Comparative Fit Index (CFI)	0.995	0.992
Tucker-Lewis Index (TLI)	0.993	0.991
RMSEA	0.02	0.023
RMSEA IC 95%	0.012–0.031	0.016–0.030

*Model 1 *Victimization (DVQ-RV), *Model 2 *Perpetration (DVQ-RP)

The results of the multivariate normality analysis for Model 1 (Victimization, DVQ-RV) showed that Mardia’s skewness and kurtosis coefficients were significantly elevated (*skewness* = 637, *χ*^2^ = 86,950, *p* < 0.001; kurtosis = 1706, *z* = 610, *p* < 0.001), indicating a clear deviation from multivariate normality. This suggested that the data did not meet the assumptions required for analyses such as confirmatory factor analysis using standard maximum likelihood estimates, which could have affected the validity of the results. To address this limitation, it was recommended to consider data transformations or robust methods such as *WLSMV*.

The multivariate normality analysis for Model 2 (Perpetration, DVQ-RP) showed that Mardia’s skewness and kurtosis coefficients were significantly elevated, with a skewness coefficient of 531 (*χ*^*2*^ = 72,518, *p* < 0.001) and a kurtosis coefficient of 1571 (*z* = 546, *p* < 0.001), indicating a significant deviation from multivariate normality in both aspects. This suggested that the data exhibited skewness and longer tails or more pronounced peaks than a multivariate normal distribution, which could have affected the validity of analyses based on standard maximum likelihood estimation.

The data in Table 4 provides a detailed analysis of the estimated parameters for both the original version of the questionnaire (DVQ-RV) and its parallel version (DVQ-RP) across five key factors: Physical, Sexual, Humiliation, Detachment, and Coercion. The table offers insights into the performance of each item within these factors, as well as overall indicators of reliability and validity.

**Table 4:** Estimated parameters for DVQ-RV and DVQ-RP factors

**Factor**	**Items**	**DVQ-RV** (Victimization)				**DVQ-RP** (Perpetration)				
		**Estimate**	**α**	**ω**	**AVE**	**Items**	**Estimate**	**α**	**ω**	**AVE**
**F1.** Physical	Cuv3a	0.87	.882	.847	.607	Cuv3b	0.54	.723	.781	.480
	Cuv7a	0.86				Cuv7b	0.68			
	Cuv10a	0.90				Cuv10b	0.94			
	Cuv11a	0.35				Cuv11b	0.53			
**F2.** Sexual	Cuv2a	0.92	.762	.747	.466	Cuv2b	0.77	.814	.890	.582
	Cuv6a	0.87				Cuv6b	0.80			
	Cuv14a	0.35				Cuv14b	0.73			
	Cuv18a	0.37				Cuv18b	0.75			
**F3.** Humiliation	Cuv9a	0.86	.748	.713	.404	Cuv9b	0.75	.765	.700	.459
	Cuv12a	0.71				Cuv12b	0.51			
	Cuv19a	0.39				Cuv19b	0.75			
	Cuv20a	0.47				Cuv20b	0.67			
**F4.** Detachment	Cuv4a	0.54	.742	.742	.421	Cuv4b	0.60	.725	.754	.413
	Cuv8a	0.66				Cuv8b	0.73			
	Cuv15a	0.74				Cuv15b	0.52			
	Cuv16a	0.64				Cuv16b	0.70			
**F5.** Coercion	Cuv1a	0.73	.767	.775	.479	Cuv1b	0.69	.864	.882	.635
	Cuv5a	0.72				Cuv5b	0.82			
	Cuv13a	0.40				Cuv13b	0.75			
	Cuv17a	0.84				Cuv17b	0.91			

*α* Alpha Ordinal, *ω* McDonald’s, *AVE* Average Variance Extracted, *DVQ-RV* Victimization, *DVQ-RP* Perpetration

In the Physical factor (F1), the DVQ-RV version demonstrates strong factor loadings, particularly with items Cuv3a (0.87), Cuv7a (0.86), and Cuv10a (0.90), leading to high reliability with an alpha of 0.882 and an omega of 0.847. The AVE of 0.607 further indicates that a significant portion of the variance in these items is captured by the factor. Comparatively, the DVQ-RP version shows slightly lower factor loadings, especially for Cuv3b (0.54) and Cuv11b (0.53), resulting in a lower alpha of 0.723 and an AVE of 0.480, suggesting that while the parallel version is reliable, it may not capture the Physical factor as robustly as the original.

For the Sexual factor (F2), both versions show strong performance. The DVQ-RV version includes high loadings for items like Cuv2a (0.92) and Cuv6a (0.87), resulting in an alpha of 0.762 and an omega of 0.747. However, the AVE is slightly below 0.5 at 0.466, indicating that there’s room for improvement in capturing the variance. On the other hand, the DVQ-RP version excels with strong loadings across all items, particularly Cuv2b (0.77) and Cuv6b (0.80), yielding a higher alpha of 0.814 and an omega of 0.890. The AVE of 0.582 is well above 0.5, suggesting that this version effectively captures the variance in the Sexual factor, making it a highly reliable and valid measure.

In the Humiliation factor (F3), the DVQ-RV version has respectable loadings, with Cuv9a at 0.86 and Cuv12a at 0.71, leading to an alpha of 0.748 and an omega of 0.713. However, the AVE is 0.404, indicating that the factor does not capture as much variance as desired. The DVQ-RP version performs slightly better, with more consistent loadings, particularly for Cuv9b (0.75) and Cuv19b (0.75), resulting in a similar alpha of 0.765 but with a slightly improved AVE of 0.459, suggesting better variance capture.

For the Detachment factor (F4), both versions perform adequately. The DVQ-RV version shows moderate loadings, with Cuv8a at 0.66 and Cuv15a at 0.74, resulting in an alpha of 0.742 and an AVE of 0.421. The DVQ-RP version shows comparable results, with Cuv8b at 0.73 and Cuv16b at 0.70, yielding an alpha of 0.725 and an AVE of 0.413. These results indicate that while both versions reliably measure the Detachment factor, the variance captured could be improved.

Finally, the Coercion factor (F5) shows strong performance in both versions. The DVQ-RV version includes high loadings for items like Cuv17a (0.84) and Cuv1a (0.73), leading to an alpha of 0.767 and an omega of 0.775. The AVE of 0.479 is close to 0.5, suggesting good variance capture. The DVQ-RP version, however, performs exceptionally well with high loadings, particularly for Cuv17b (0.91) and Cuv5b (0.82), resulting in a high alpha of 0.864 and an omega of 0.882. The AVE of 0.635 indicates that this version captures a substantial portion of the variance in the Coercion factor, making it a very robust measure.

Overall, both the DVQ-RV and DVQ-RP versions show strong psychometric properties, with the DVQ-RP version demonstrating particularly high reliability and validity in the Sexual and Coercion factors. While the original DVQ-RV version generally performs well, the parallel version offers slight improvements in capturing variance for certain factors, making it a strong alternative for measuring dating violence.

**Fig. 1:**
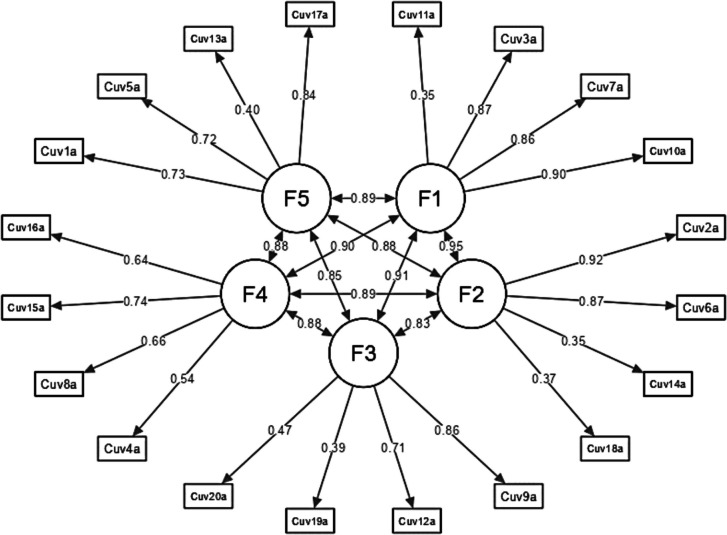
Victimization model 1 (DVQ-RV). F1 = Physical, F2 = Sexual, F3 = Humiliation, F4 = Detachment, F5 = Coercion

The victimization model (Fig. 1) illustrates various dimensions and mechanisms related to the experiences of those affected, comprising five key factors: Physical, Sexual, Humiliation, Detachment, and Coercion. The Physical factor involves direct forms of victimization such as harm or assault, including acts like hitting, which result in visible injuries and psychological trauma, often with immediate effects on health. The Sexual factor encompasses victimization related to sexual abuse or coercion, including unwanted advances or harassment, leading to profound emotional and psychological effects and long-term trauma. Humiliation involves actions aimed at belittling or degrading the victim, such as verbal abuse or public shaming, which can erode self-esteem and mental health, causing feelings of worthlessness and anxiety. Detachment refers to the psychological or emotional separation from the trauma, which may help manage overwhelming emotions but can also hinder forming healthy relationships. Coercion involves using threats or manipulation to control the victim, including blackmail or intimidation, undermining autonomy and perpetuating a cycle of abuse and control. Together, the victimization model provides a comprehensive view of how these different factors interact and impact the individual’s physical, emotional, and psychological well-being. Understanding these factors is crucial for developing effective interventions and support mechanisms for victims.

**Fig. 2:**
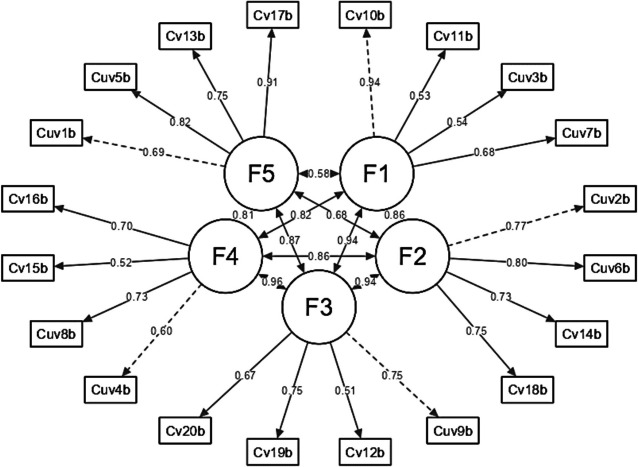
Perpetration model (DVQ-RP). F1 = Physical, F2 = Sexual, F3 = Humiliation, F4 = Detachment, F5 = Coercion

The perpetration model (Fig. 2) depicted in the figure outlines the different dimensions and mechanisms involved in perpetrating victimization, featuring five key factors: Physical, Sexual, Humiliation, Detachment, and Coercion. Physical perpetration includes acts of violence such as hitting or pushing, directly affecting the victim’s physical health. Sexual perpetration involves unwanted sexual advances or assault, leading to severe emotional trauma. Humiliation entails degrading behaviors like verbal abuse that undermine the victim’s self-esteem. Detachment refers to the perpetrator’s lack of empathy or emotional involvement, allowing them to harm the victim without acknowledging the consequences. Coercion involves using threats or manipulation to control the victim, perpetuating a cycle of abuse. Together, these factors illustrate the various ways in which perpetrators inflict harm and control over their victims.

The Heterotrait-Monotrait (HTMT) ratio of correlations for the victimization model illustrates the degree of interrelation among its factors, providing insight into their discriminant validity. Strong correlations are observed between factors such as F1 (Physical) and F3 (Humiliation) with a value of 0.766, and F1 and F5 (Coercion) with a value of 0.779, indicating significant relationships between these dimensions. In contrast, the relationship between F2 (Sexual) and F4 (Detachment) is weaker, with an HTMT ratio of 0.552, suggesting a clearer distinction between these factors.

Overall, the model’s factors are interrelated to varying degrees, with some showing notably strong associations, highlighting both overlap and unique aspects within the realm of victimization.

For the perpetration model in dating violence, the HTMT ratio of correlations reveals the level of discriminant validity among its factors. Most correlations are strong, reflecting significant interrelationships. For instance, the correlation between F1 (Physical) and F2 (Sexual) is 0.751, and between F1 and F3 (Humiliation) is 0.756, indicating strong associations. Similarly, F1’s relationships with F4 (Detachment) and F5 (Coercion) are 0.762 and 0.759, respectively, suggesting considerable overlap. Additionally, F2 (Sexual) and F3 (Humiliation) have a correlation of 0.723, while F2 and F4 (Detachment) are at 0.678, both showing strong to moderate associations. The correlation between F2 and F5 (Coercion) is 0.717. Strong associations are also seen between F3 (Humiliation) and F4 (Detachment) at 0.749, and between F3 and F5 (Coercion) at 0.762. The highest correlation is between F4 (Detachment) and F5 (Coercion), at 0.786, indicating the strongest relationship among the factors.

Regarding the invariance of the Dating Violence Questionnaire, both for Victimization and Perpetration, the results indicated that the measure was invariant across gender. Specifically, the Comparative Fit Index (CFI) and the Tucker-Lewis Index (TLI) were both below 0.90, and the root mean square error of approximation (RMSEA) was above 0.08. The AIC (7584–7975) and BIC (7823–7975) results in the psychometric models of the Dating Violence Questionnaire for Victimization and Perpetration (DVQ-VP) in the Ecuadorian population reflect evaluations of different factorial structures, where lower values indicate a better balance between fit and simplicity.

These findings suggest that the questionnaire’s factor structure and measurement properties vary across different demographic groups, which could impact the reliability and validity of the instrument when used with diverse populations.

**Fig. 3:**
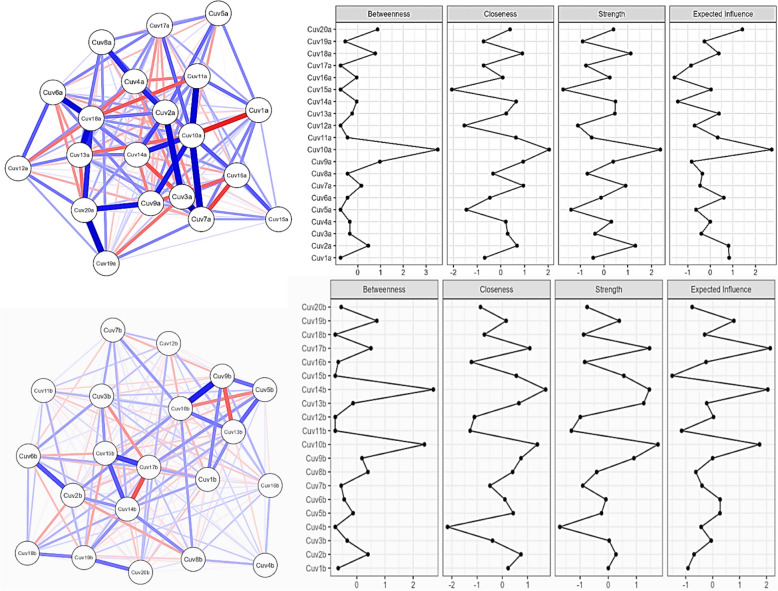
Network analysis of DVQ-RV and DVQ-RP items

In the Betweenness plot (Fig. 3), which measures how often an item acts as a bridge along the shortest path between other items, items Cuv20a, Cuv19a, and Cuv18a show high values, indicating they play crucial roles as intermediaries in the network. On the other hand, items Cuv3a, Cuv2a, and Cuv1a have the lowest betweenness values, suggesting they are less central in terms of connecting other items.

In the Closeness plot, which measures how close an item is to all other items in the network, items Cuv6a, Cuv14a, and Cuv19a have high closeness values, indicating they are closer to other items in the network.

In contrast, items Cuv1a, Cuv3a, and Cuv10a have lower closeness values, suggesting they are more peripheral. In the Strength plot, which measures the sum of the weights of connections an item has with other items, items Cuv18a, Cuv17a, and Cuv10a have high strength values, indicating strong connections with other items. Meanwhile, items Cuv1a, Cuv2a, and Cuv13a show lower strength values, suggesting weaker overall connections.

Finally, in the Expected Influence plot, which measures the impact an item has on other items in the network, items Cuv20a, Cuv19a, and Cuv18a have high expected influence values, indicating they have a significant impact on other items. In contrast, items Cuv1a, Cuv2a, and Cuv3a have lower expected influence values, suggesting they have less impact on other items.

Overall, items Cuv20a, Cuv19a, and Cuv18a appear to be the most central and influential in the network, while items Cuv1a, Cuv2a, and Cuv3a are less central and influential (Fig. 3).

Table 5 presented the correlations between the various measures of the DVQ-VP, categorized into Victimization and Perpetration. For Victimization, the correlations were as follows: Physical Victimization correlated with Sexual Victimization at 0.515, Humiliation at 0.547, Detachment at 0.396, and Coercion at 0.457. Sexual Victimization correlated with Humiliation at 0.435, Detachment at 0.350, and Coercion at 0.411. Humiliation correlated with Detachment at 0.415 and Coercion at 0.466. Detachment correlated with Coercion at 0.438.

**Table 5:** Correlations of the DVQ-VP measures

	**1**	**2**	**3**	**4**	**5**	**6**	**7**	**8**	**9**	**10**
**Victimization**										
**1.** Physical	1									
**2.** Sexual	.515	1								
**3.** Humiliation	.547	.435	1							
**4.** Detachment	.396	.350	.415	1						
**5.** Coercion	.457	.411	.466	.438	1					
**Perpetration**										
**6.** Physical	.361	.349	.479	.525	.402	1				
**7.** Sexual	.542	.535	.488	.395	.440	.501	1			
**8.** Humiliation	.507	.403	.557	.415	.533	.462	.550	1		
**9.** Detachment	.456	.401	.473	.491	.572	.494	.587	.495	1	
**10.** Coercion	.464	.605	.430	.369	.420	.368	.485	.462	.522	1

^***^p <.001 in all the cases

For perpetration, the correlations were as follows: Physical Perpetration correlated with Sexual Perpetration at 0.361, Humiliation at 0.479, Detachment at 0.525, and Coercion at 0.402. Sexual Perpetration correlated with Humiliation at 0.488, Detachment at 0.395, and Coercion at 0.440. Humiliation correlated with Detachment at 0.415 and Coercion at 0.533. Detachment correlated with Coercion at 0.572.

The correlations between Victimization and Perpetration measures were also noted. Physical Victimization correlated with Physical Perpetration at 0.361, Sexual Perpetration at 0.542, Humiliation at 0.507, Detachment at 0.456, and Coercion at 0.464. Sexual Victimization correlated with Physical Perpetration at 0.349, Sexual Perpetration at 0.535, Humiliation at 0.403, Detachment at 0.401, and Coercion at 0.605. Humiliation Victimization correlated with Physical Perpetration at 0.479, Sexual Perpetration at 0.488, Humiliation at 0.557, Detachment at 0.473, and Coercion at 0.430. Detachment Victimization correlated with Physical Perpetration at 0.525, Sexual Perpetration at 0.395, Humiliation at 0.415, Detachment at 0.491, and Coercion at 0.369. Coercion Victimization correlated with Physical Perpetration at 0.402, Sexual Perpetration at 0.440, Humiliation at 0.533, Detachment at 0.572, and Coercion at 0.420. All these correlations were significant at *p* < 0.001, indicating strong relationships between these variables.

## Discussion

The current research independently evaluated the measurement models of the DVQ-RV/DVQ-RP and examined the model based on the interdependence approach for the Dating Violence Questionnaire for Victimization and Perpetration (DVQ-VP), which includes reports on victimization and perpetration from 819 young Ecuadorians.

This study has provided valuable insights into the factorial structure and reliability of the DVQ-RV scale (Rodríguez-Díaz et al., 2017; Rodríguez-Franco et al., 2022), designed to assess victimization in romantic relationships within the Ecuadorian context, where there is a lack of validated and reliable instruments for this purpose. The DVQ-R, through its five factors, covers the three main categories of partner violence identified in the literature (Rodríguez-Díaz et al., 2017), with a parsimonious structure and adequate length for applied research. Consistent with previous studies in the Hispanic American context (Alfaro-Urquiola, 2020; Lara and López-Cepero, 2018; Rodríguez-Díaz et al., 2017) and other contexts (López-Cepero et al., 2015; Presaghi et al., 2015), the DVQ-R has demonstrated good psychometric properties in young Ecuadorian women.

In terms of factorial validity, the original five-factor correlated model proposed by Rodríguez Díaz et al. (2017) showed a good fit to the data. Unlike the original version, it was not necessary to include covariances between some items. The reliability indices obtained were also satisfactory, with values between ω > 0.70 and ω > 0.70. Although these values may appear higher than those in previous studies, it is important to note that a different reliability index (ordinal omega) was used instead of Cronbach’s alpha, making direct comparison impossible.

These results represent a significant advance in research on intimate partner violence (IPV) in Ecuador and provide a measurement tool for partner victimization with demonstrated validity and reliability, which previously did not exist in this context. This allows for continued efforts to combat IPV, aligning with the social plans of the Comprehensive Organic Law to Prevent and Eradicate Violence against Women in Ecuador, and offers a scientific instrument to evaluate and analyze the reality of IPV efficiently and accurately.

A similarity with the original work by Rodríguez Díaz et al. (2017) is the reported mean levels of different forms of victimization. As in the Spanish sample, Ecuadorian women reported higher mean levels of detachment, coercion, humiliation, sexual violence, and physical violence, respectively. These results, supporting the findings of Rodríguez-Díaz et al. (2017), are consistent with studies in this field, highlighting the higher prevalence of psychological IPV (detachment, coercion, and humiliation) compared to physical and sexual IPV. This underscores the need to consider psychological IPV as more than just a precursor to physical IPV and to analyze it specifically (Juarros-Basterretxea et al., 2018, 2019, 2020; Romero-Martínez et al., 2023).

The research confirms the validity of the DVQ-RV for measuring partner victimization, replicating findings in other contexts. However, it is relevant to note that this version of the DVQ-RV presents a significant variation from previous versions, specifically in the Likert scale response options. In its original version, the DVQ-RV used a five-option Likert scale from 0 to 4, labeled as never, sometimes, frequently, usually, and all the time (Juarros-Basterretxea et al., 2018). Although these labels reflect an increase in victimization, they could be interpreted subjectively by the participants, affecting the measurement (e.g., if the higher categories are underrepresented or considered equal). To address this potential issue, the current version has been modified to use a response format that explicitly refers to the number of times, commonly used in measuring violence. Thus, the Likert scale now ranges from 0 to 4, but the options are labeled as never or zero times, once, twice, three times, and four or more times. This modification introduces a new component of objectivity to the DVQ-RV and allows for homogenizing participants’ responses, avoiding potential biases arising from differential interpretation of terms like frequently versus usually (Alfaro-Urquiola, 2020). Furthermore, the study highlights significant variations in the experiences and perceptions of dating violence across different age groups and between genders, emphasizing the need to consider these differences when analyzing the prevalence and impact of victimization (Rodríguez-Franco et al., 2022).

### Limitations

The current research presents both strengths and potential limitations. This study enhances knowledge about a valid and reliable assessment tool by supporting the results obtained in previous research (Rodríguez-Díaz et al., 2017) and, to our knowledge, it is the first study to report evidence on the reliability (internal consistency) and validity (factor structure) of a DV/IPV measurement instrument that considers the interdependence between perpetration and victimization reports among a sample of young Ecuadorians. The inclusion of the interdependent nature of DV victimization and perpetration (Herrero et al., 2020) is an innovative approach that contributes to the advancement of the field by presenting a useful and concise evidence-based perpetration-victimization model, often underrepresented in many studies (Exner-Cortens et al., 2016).

Nevertheless, the DVQ-VP has the inherent limitations of behavioral measurement instruments (Hardesty & Ogolski, 2020). It would be beneficial for future research to use it alongside other instruments and data collection methods and to assess its convergent and divergent validity considering different levels of IPV correlates (Dardis et al., 2016; Hammock et al., 2017; Herrero et al., 2016, 2018; Juarros-Basterretxea et al., 2020). Additionally, it would be advisable to include other sources of information and methods to assess actual injuries (Vilarino et al., 2018) and verify victimization and perpetration (Gancedo et al., 2021). Furthermore, future research should explore the use of second-order factor analysis models, as this approach could shed light on the hierarchical structure of the constructs involved, offering a more comprehensive understanding of the relationships between primary and secondary factors. This type of analysis may help researchers to better capture the multifaceted nature of IPV and improve the precision of theoretical and practical applications of the instrument.

In addition, the analysis of measurement invariance warrants further attention, as the findings suggest that the DVQ-VP is not invariant across gender and age groups. This is a significant limitation that impacts the comparability of results between these groups. Future studies should delve deeper into the methodological approach used to assess invariance, including testing for configural, metric, and scalar invariance, and provide a thorough discussion of the implications of non-invariance on data interpretation. Non-invariance indicates that the instrument may measure different constructs or attributes depending on the demographic group, which could limit its applicability and fairness. Addressing these limitations is essential for ensuring the instrument’s robustness and its ability to provide reliable data across diverse populations.

Finally, incorporating qualitative methods, such as in-depth interviews or focus groups, alongside the DVQ-VP could provide richer insights into the lived experiences of victims and perpetrators. Such methods could complement the quantitative data and help contextualize the findings, particularly in understanding cultural and social factors that may influence IPV dynamics. Cross-cultural adaptations and validations of the instrument are also necessary to ensure that it remains relevant and accurate when applied to populations with diverse sociocultural characteristics. By addressing these limitations and expanding the scope of research, the DVQ-VP can continue to evolve as a valuable tool for studying IPV.

Moreover, the sample of the current research also represents a significant strength. First, all participants were involved in an intimate relationship at the time of the study, making the data more reliable than those from studies based on past or recent relationships due to reduced recall bias. Second, the sample size of the study exceeds those of other studies (Exner Cortens et al., 2016), especially considering that the sample consisted of current couples. However, it is also true that the sample was not representative, so any generalization of the data should be made with caution. For example, generalized and specific perpetrators were not included (Cantos et al., 2019). Similarly, although the number of participating couples was high, all were heterosexual couples, and future research should replicate these results considering greater diversity among couples (Harden et al., 2020; Laskey et al., 2019; Lin et al., 2020; Martínez-Gómez et al., 2021; Peitzmeier et al., 2020; Ramiro-Sánchez et al., 2018; Rojas-Solís et al., 2019).

## Conclusion

In conclusion, the DVQ-VP stands out as the only dating violence (DV) measurement instrument that considers the interdependence between reports of perpetration and victimization, validated for use among young Ecuadorian participants regardless of gender. This innovative approach of including interdependence in self-reported scores represents a significant methodological improvement in the assessment of dating violence. It allows for more accurate measurement and a deeper understanding of violence, capturing the complex and bidirectional link between victimization and perpetration.

The DVQ-VP, like its predecessor the DVQ-RV, has proven to be a valid, reliable, and relatively brief measurement instrument. This makes it a potentially useful tool for applications in young community samples. Its compact and efficient design is suitable for the primary detection of dating violence in terms of both perpetration and victimization. The DVQ-VP’s ability to capture the interrelated dynamics of these experiences positions it as a valuable tool for researchers and professionals in the fields of mental health and intimate partner violence intervention. Additionally, its use can facilitate early identification and timely addressing of violence situations, thereby contributing to the prevention and support of affected youth.

For future research on the psychometric properties of the Dating Violence Questionnaire for Victimization and Perpetration (DVQ-VP), it is essential to conduct a multigroup analysis to determine factorial invariance by sex and age. This procedure, using techniques such as confirmatory factor analysis (CFA), helps verify whether the structure of the psychometric model is consistent across different groups. It is recommended to evaluate configurational, metric, and scalar invariance to ensure that group comparisons are valid and accurate.

Another key aspect is the incorporation of nomological validity (NV), which assesses the association of the measured construct with others that are part of related theories (Spiro et a., 1990). To achieve this, correlational or experimental studies should be designed to include variables such as anxiety, depression, self-esteem, or relationship satisfaction. This will confirm whether the theoretically expected relationships are reflected in the results obtained with the DVQ-VP. Methods such as structural equation modeling (SEM) or regression analysis can be useful for this purpose, analyzing the strength and direction of these associations.

Additionally, it is recommended to increase the diversity of samples in future studies. Including participants from different cultural, socioeconomic, and geographical contexts will ensure that the results are generalizable and representative of various populations. Similarly, it is suggested to compare the DVQ-VP with other similar instruments to evaluate its incremental validity and determine whether this questionnaire provides additional value in measuring dating violence.

Finally, conducting longitudinal studies would be ideal to assess the temporal stability of the instrument and its predictive capacity over time. It is also recommended to perform a differential item functioning (DIF) analysis to detect potential biases that may favor or disadvantage certain groups, such as those based on sex or age. These efforts will not only strengthen the validity and reliability of the DVQ-VP but also contribute to the development of a robust and useful instrument for studying dating violence.

## Data Availability

The data used in this study are stored and managed by the corresponding author, who can be contacted for any inquiries. Due to ethical considerations, the data are not publicly available, as the researchers are committed to protecting participant privacy and ensuring that the data are used solely for academic purposes.

## Abbreviations

DVQ-VP: Dating Violence Questionnaire for Victimization and Perpetration
DVQ-RV: Dating Violence Questionnaire for Victimization
DVQ-RP: Dating Violence Questionnaire for Perpetration
AVE: Average variance extracted
CFI: Comparative Fit Index
TLI: Tucker-Lewis Index
NNFI: Bentler-Bonett Non-normed Fit Index
RNI: Relative Noncentrality Index
NFI: Bentler-Bonett Normed Fit Index
RFI: Bollen’s Relative Fit Index
IFI: Bollen’s Incremental Fit Index
